# Research progress on the mechanism by which skin macrophage dysfunction mediates chronic inflammatory injury in diabetic skin

**DOI:** 10.3389/fendo.2022.960551

**Published:** 2022-08-24

**Authors:** Jiali Huang, Shili Zhang, Xinyi Ding, Shuxian Li, Xiangrong Luo, Ying Cao, Fang Gao, Mengchen Zou

**Affiliations:** ^1^ Department of Endocrinology and Metabolism, Nanfang Hospital, Southern Medical University, Guangzhou, China; ^2^ School of Public Health and Tropic Medicine, Southern Medical University, Guangzhou, China

**Keywords:** macrophage dysfunction, chronic skin inflammation injury mechanism, diabetic skin lesions, diabetic foot, pathogenesis

## Abstract

Macrophages, the main immune cells in the skin, form an innate immune barrier. Under physiological conditions, skin maintains immune barrier function through macrophage phagocytosis and antigen presentation. Parenchymal and stromal cell regeneration plays an important role in skin injury repair and uses macrophage plasticity to influence and stabilize the skin microenvironment. Diabetic skin lesions are the most common diabetes complication and are involved in the early pathophysiology of diabetic foot. Therefore, studying the initial link in diabetic skin lesions is a research hot spot in the early pathogenesis of diabetic foot. Skin inflammation caused by hyperglycaemia, oxidative stress and other injuries is an important feature, but the specific mechanism is unknown. Recent studies have suggested that chronic inflammatory injury is widely involved in a variety of skin diseases, and whether it plays an important role in diabetic skin lesions is unclear. In this review, current research hotspots were combined with the pathogenesis of diabetic skin lesions and analysed from the perspectives of the physiological function of skin macrophages, the impairment of skin macrophages in diabetes, and the mechanism of chronic inflammatory injury in macrophages to provide a theoretical basis for early screening and evaluation of diabetic foot.

## Introduction

Diabetes is a costly disease, and nearly one-third of the costs are attributable to the management of diabetic foot disease. Amputation of diabetic patients ranks first among nontraumatic amputations and has become a major public health problem that places a heavy burden on society (1, 2). It is widely believed that diabetic foot (DF) refers to foot infection, ulcers, and/or deep tissue destruction caused by nerve abnormalities in the distal lower extremity and peripheral vascular lesions to varying degrees. Traditional thinking has ignored the changes in the functional state of the skin before the appearance of ulcers (3, 4). In recent years, many studies have suggested that diabetic dermatopathy (DD) may be an important part of the early pathogenesis of DF. DDs can be divided into microvascular DDs, neurogenic DDs, metabolic disorders, DDs, diabetic skin infections and hypoglycaemic drug-induced skin reactions according to different aetiologies. These lesions can manifest as skin infection, inflammation, xerosis and DF (5).

The pathogenesis of DDs has not been fully elucidated. Some scholars believe that DD pathogenesis includes a local hyperglycaemic environment and the accumulation of glycation end products, growth factor starvation theory, microvascular damage, and excessive repair cell apoptosis (6). In recent years, with improvements in inspection technology, many scholars have begun to propose that subclinical skin inflammation (skin inflammation occurring in the absence of any visual inflammation) is a key link in promoting the occurrence and development of DDs (6, 7). This concept has led to a reassessment of the triggers of DD development, focusing on the role of inflammation and poor immune system regulation in the pathogenesis of DDs. This review will discuss the sources of skin macrophages, five physiological functions, chronic inflammatory injury in macrophages and the pathogenesis of subclinical diabetes dermatosis.

## Origin and physiological function of skin macrophages

Macrophages are the main immune cells in the skin and are mainly distributed in the superficial dermis. It is now widely believed that dermal macrophages are produced in the yolk sac and foetal liver during embryonic development (8). In adulthood, these cells are replaced by haematopoietic stem cells (9). When tissue homeostasis is disturbed, bone marrow-derived monocytes are recruited from the blood to the affected site, where they differentiate into macrophages (9, 10). Wounds, minor injuries and minor obstacles can open the way for skin invasion (11). Therefore, preventing pathogen invasion and tissue repair are key processes in the successful regeneration of the skin barrier, as well as the theoretical basis for wound healing. Skin macrophages have functions such as chemotaxis, plasticity, phagocytosis, intracellular signalling and the ability to secrete cytokines (12). These abilities enable skin macrophages to maintain skin homeostasis through chemotactic movement, the phagocytosis of pathogens, killing target cells (ageing and dead cells), and immune regulation (12).

### Skin macrophage patrols

Similar to other tissue-resident macrophages, skin macrophages are inactive. During physiological conditions, these cells patrol the local environment and keep our tissues free of debris. However, the ability of the body to maintain immune homeostasis despite the constant exposure of the skin to bacteria and air suggests that skin macrophages may be more dynamic than expected. During homeostasis, tissue-resident macrophages act as sentinels to dispose of pathogens. However, once the skin is infected with a highly pathogenic organism, macrophages have highly proinflammatory properties and start recruiting more macrophages and other immune cells. When stimulated by pathogen-associated molecular patterns (PAMPs) (released from invading pathogens) and damage-associated molecular patterns (DAMPs) (released from damaged or dead cells in response to infection and injury), macrophages can direct their pseudopods along concentration gradients of certain chemicals (chemokines) to the site where these substances are released. This property is called chemotactic motility. Excessive chemotactic movement results in macrophage osmosis, which does not seem beneficial, considering the effector functions of these cells. Tissue penetration of macrophages may delay tissue death, but it causes serious skin inflammation.

### Plasticity of skin macrophages

The different roles of macrophages are controlled by their robust plasticity. Macrophages respond to external factors from the tissue microenvironment by activating different intracellular pathways that lead to specific polarization patterns. At present, macrophages are roughly classified as classically activated/M1 macrophages or selectively activated/M2 macrophages according to their responses to different stimuli. M1 macrophages and M2 macrophages are considered to preferentially trigger Th1-type and Th2-type responses (13, 14), representing proinflammatory and anti-inflammatory polarization states, respectively. These classifications show that macrophages exist on a continuum, and macrophage phenotype can be better described as a series of grades over a wide range (15). Therefore, macrophages are further divided into M2a, M2b, M2c and M2d according to the molecules that lead to the activation of M2 macrophages and their gene expression profiles (16). Macrophages differ in their expression of cell surface markers, cytokine secretion and biological functions (17).

The polarization state of skin macrophages plays an important and positive role in the maintenance of skin homeostasis. The polarization of skin macrophages has profound effects on various physiological and pathological conditions, such as angiogenesis, wound repair, inflammation and tumour formation (18). Upon exposure to the local microenvironment, macrophages convert into one of two phenotypes (M1 and M2) by activating relevant signalling pathways. Two signals, interferon gamma (IFN-γ) and lipopolysaccharide (LPS), are mainly involved in M1 polarization (13). After binding to the corresponding receptors (interferon gamma receptor (IFNGR) and toll-like receptor 4 (TLR4)), IFN-γ and LPS recruit Janus kinase 1/2 (JAK1/2) and the TLR domain-containing adaptor proteins (TIR domain-containing adaptor inducing interferon beta (TRIF)) and myeloid differentiation factor 88 (MyD88). Further activation of the downstream factor kappa B kinase (IKK-β), interferon regulatory factor 3 (IRF3), IL-1 receptor-associated kinase 4 (IRAK-4), TNF receptor-associated kinase 6 (TRAF-6) and nuclear factor inhibitors results in the activation of signal sensor and transcription 1 (STAT1) and nuclear factor kappa B (NF-κ B) (19). These factors lead to M1-type macrophages that promote the expression of inflammatory genes, including tumour necrosis factor α (TNF-α), B-cell-activating factor (BAFF), IL-1β, cyclooxygenase 2 (COX2), chemokine (C-X-C motif) ligand 9 (CXCL9), CXCL10, IL-6 and IL-12 (20, 21). On the other hand, IL-4, IL-10 and IL-13 bind to corresponding receptors and activate JAK1/3, signal transducer and activator of transcription 3 (STAT3) and STAT6, respectively (22). Activated STAT3 and STAT6 promote M2 polarization and cause the production of anti-inflammatory cytokines.

When external substances contact or enter the skin, macrophages recognize them, transmit information and memory, phagocytose and reject substances, and produce specific cell components, cytokines, immunoglobulin and complement *in vivo* to produce an inflammatory response. This response is not only a self-protection mechanism in the body but is also the mechanism of many skin diseases. In the last five to ten years, macrophage polarization has become an area of concern. Imbalanced skin macrophage polarization is involved in the pathogenesis of a variety of skin lesions. This includes the M1-ylation of psoriatic skin macrophages and M1-ylation of systemic lupus erythematosus skin macrophages (23).

### Skin macrophages as killers

Skin macrophages have strong phagocytic and killing abilities and are important immune cells that are involved in nonspecific immune defence in the body. In a broad sense, phagocytosis refers to the elimination of invading pathogens by removing tissue debris after injury and apoptotic cells during development. However, to distinguish the necrotic function of macrophages, the phagocytic function specifically refers to the process by which macrophages remove foreign microorganisms. When pathogenic microorganisms or other foreign antigens enter the body, macrophages interact with their surface-specific receptors (Fc gamma R, CR3) and exogenous particles (proteins, such as antibodies, complement proteins, lectins), causing the particles to migrate to the cell surface, and local actin cytoskeleton aggregation and pseudopodia formation occurs. After macrophages completely phagocytose granules, actin depolymerizes and is transported by vesicles to promote the maturation of phagosomes. After lysis, actin fuses with lysosomes to form phagolysosomes, which are digested and decomposed by various hydrolases and enzymes in lysosomes (23). The most important changes in phagocytosis are those in the cytoskeleton. Cell membrane Rho family GTP enzymes transmit signals to facilitate cytoskeletal changes during phagocytosis (24). It has been reported that messenger molecules such as phosphoinositide 3-kinase (PI3-K), cAMP, cGMP and PKC are involved in the regulation of nonspecific phagocytosis by macrophages (25, 26).

### Skin macrophages targeting dead cells

Unlike macrophage phagocytosis, cytolysis is the process by which macrophages remove apoptotic cells. Cytokinesis is similar to macropinocytosis in that RhoA activity is inhibited by receptors that recognize apoptotic cells, while Rac1 activation assists macrophages in taking up apoptotic bodies (27). Actin polymerization makes the cells extend its pseudopodia, and then the cell membrane is invaginated to form membrane folds that wrap the apoptotic cells and internalize them into efferosomes. The fusion of lysosomes introduces hydrolase into the efferosome, forming a harsh acidic environment and destroying apoptotic cells (27).

The process of necroptosis is a closely regulated process (28), including a “discover me”, in which the apoptotic cell exposes phosphatidylserine (PS), releases cytokines, and attracts macrophages to the apoptotic cell region. “Catch me” signals include macrophage overexpression of intercellular adhesion molecule (ICAM) and the scavenger receptors CD36 and CD14, and these cells interact with apoptotic cells through these molecules. “Eat me” signals involve macrophages destroying apoptotic cells by forming burial bodies (29). Class B Type I scavenger receptors induce the activation of the RhoGTP enzyme Rac1 by recognizing PS, and activated Rac1 leads to the rearrangement of cytoskeletal actin. Kimura et al. (30) and Martin et al. (31) found that apoptotic cells upregulated the expression of monocyte chemotactic protein (MCP3) by releasing uridine diphosphate (UDP), thus promoting the infiltration of macrophages into necrotic areas, which may indirectly increase the clearance of apoptotic cells by macrophages.

### Secretory abilities of macrophages

Mononuclear phagocytes are capable of synthesizing and secreting a large number of substances that can affect the state of the skin, some of which are constituent secretions that require no stimulation. However, most of the products secreted by macrophages are affected by stimuli acting on them. Generally, macrophage secretion is associated with their activation state and is closely related to their bactericidal and tumoricidal activity. Some products can also act on macrophages to regulate the function of these cells (12). Products synthesized and secreted by mononuclear macrophages include a variety of enzymes, reactive oxygen species (ROS) and some factors that regulate other cells (32). In this review, we focus on ROS and cytokines secreted by macrophages.

M1 macrophage activation induces proinflammatory cytokines, including TNF-α, IL-1α, IL-1β, IL-6, IL-12, IL-18 and IL-23. The production of nitric oxide (NO), ROS and reactive nitrogen species (RNS) (33) promotes T-helper type 1 (Th1) and Th17 responses to provide an effective mechanism for killing. M1 and M2 macrophages have different cytokine profiles, and M2 macrophages express anti-inflammatory molecules such as IL-10, TGF-β and IL-1RA to support the resolution of inflammation. M2 macrophages also express a large number of endogenous receptors, including the scavenger receptor CD163, statin 1, and the C-type lectin receptors CD 206, CD 301, Dectin-1, and CD 209 (12, 33). In addition, M2 macrophages recruit Th2, regulatory T cells (Tregs), eosinophils, and basophils by secreting the chemokines CCL 17, CCL 18, CCL 22, and CCL 24 (12).

## The mechanism of macrophage-mediated chronic inflammatory injury is involved in the subclinical inflammation of diabetic skin

**Figure 1:**
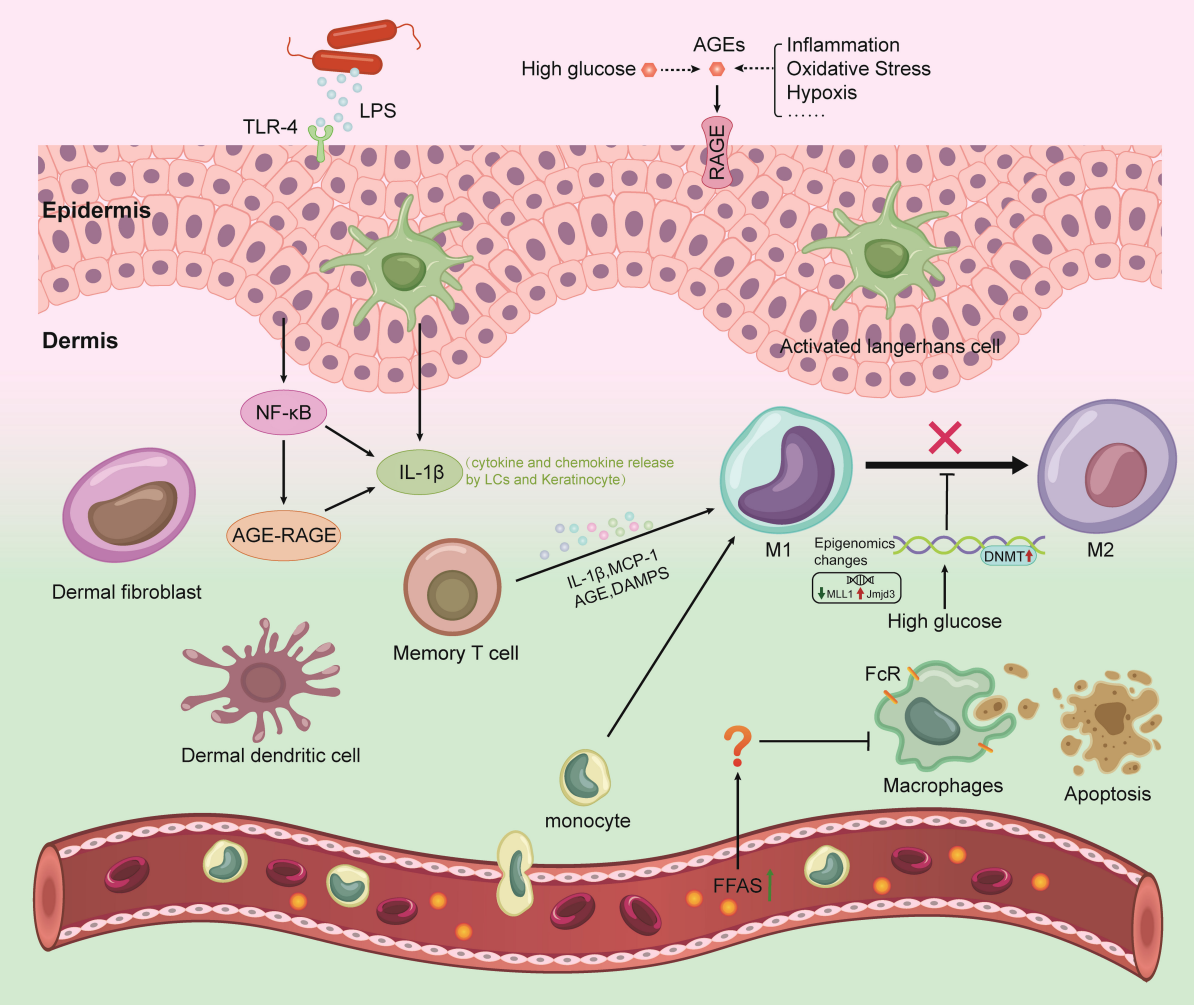
Mechanisms of the interaction between gut microbiome with skin and brain in DM. LPS, lipopolysaccharide; AGEs, advanced glycation end products; RAGE, receptor for advanced glycation end products; ROS, reactive oxygen species; TLR4, toll-like receptor 4; DAMPs, damage-associated molecular patterns; NF-κB, nuclear factor kappa B; DNMT, DNA methyltransferase; IL-1β, interleukin 1 beta; MCP-1, monocyte chemotactic protein 1; LCs, Langerhans cell.

In the past, scientists have focused on skin inflammation and the processes that lead to its cellular manifestations, but less research has focused on subclinical dermatitis. However, as testing technology has improved, the concept has gradually gained traction in the field. Subclinical skin inflammation refers to inflammation that is not detectable to the naked eye (30) and can only be detected using other techniques, such as direct observation by advanced optics, skin biopsy and histology or by measuring the indirect enhancement of biomarkers. The concept of subclinical cutaneous inflammation stems from the understanding of the relevance of innate immune detection in the skin (4). The skin, with the help of a variety of immune cells (especially macrophages), is better able to defend against harmful damage than other tissues, causing subclinical skin inflammation. In the absence of visible skin inflammation, skin macrophages are in a nonstatic state. These cells have been fighting for a long time before the body exhibits clinical symptoms, producing invisible inflammatory pathological changes (**Figure 1**). Studies have shown that such undiagnosed dermal inflammatory pathological changes are the pathological basis of skin lesions (4). A better understanding of subclinical dermatitis could help clinicians prevent acute or chronic early inflammatory processes in the skin.

As mentioned previously, macrophages normally maintain skin homeostasis through chemotactic movement, the phagocytosis of pathogens, the killing of target cells (senescent, dead cells), and immune regulation. A transient inflammatory lesion in skin tissue is beyond the scope of this review, which focuses on chronic, sustainable skin inflammation. This idea is in line with previous understanding that chronic systemic inflammation plays a key role in the pathogenesis of type 2 diabetes mellitus (T2DM) and insulin resistance. T2DM-associated inflammation is characterized by an increased number of macrophages in different tissues. The risk of T2DM complications is closely related to the increased levels of inflammatory factors such as TNF-α, IL-1β, IL-6, IL-18 and C-reactive protein (CRP) (34). It is unclear whether macrophages are involved in the development of subclinical diabetic skin inflammation.

### Increased chemotactic motility as an initiating factor

PAMPs and DAMPs initiate chemokine production in various immune cells. The chemotactic motility of macrophages is directly regulated by chemokines. Chemokines have been shown to act as signalling molecules in the inflammatory response (35) and are key signalling molecules that regulate the pathophysiological process of diabetes and its complications. Pan et al. (36) showed that the concentration of various chemokines in the plasma and serum of T2DM patients was significantly higher than that in the control group (37). This result may explain the large amount of macrophage infiltration in the diabetic mouse model. A number of studies have shown that compared with normal epidermal cells in skin tissue, which exhibited a stratified arrangement, a clear hierarchy, and orderly dermal collagen, early diabetic model mice (6 weeks) exhibit pathological changes in skin tissue, as indicated by epidermal and dermal thinning, reduced number of cells and disordered collagen (38, 39). However, less attention has been given to inflammatory cell infiltrates in the dermis of diabetic skin. In fact, there is inflammatory cell infiltration in the early stages of diabetes, and there have been many studies to support this idea. In addition, by comparing the plantar skin pathology of patients with DDs (DD group) and normal healthy people in our previous study, we found that the skin in the DD group showed significant pathological changes, such as a large amount of macrophage infiltration in the dermis and increased expression of inflammatory factors, showing the characteristic inflammatory changes in DDs. The relationship between diabetic inflammatory skin lesions and a large increase in chemokines and macrophage infiltration is unknown. However, it is clear that skin macrophages play an important role in DDs. Chemokine production may contribute to the increased infiltration of macrophages, which in turn release more inflammatory cytokines, leading to cell death, causing microvascular damage, inducing apoptosis, and damaging the protective barrier of the skin.

### The macrophage phenotype tends to be proinflammatory

Monocytes enter peripheral tissue in response to changes in tissue that the body perceives as injury. Once inside the tissue, these cells differentiate into macrophages. Macrophages are highly plastic cells with different phenotypes, depending on the surrounding microenvironment. Their main role is to engulf pathogens and dead cells, which help repair damage, and produce chemokines and cytokines to call on extra white blood cells to protect the organism when needed. Diabetic mouse models show that diabetes impedes the progression of the disease through the process of macrophage recruitment and phenotypic transition to proinflammatory macrophages. This phenomenon can be verified in a variety of diabetes complications, and macrophages are involved in the occurrence and development of diabetes complications (36). To date, there have been no studies on macrophage function or phenotype in human DDs. However, CD68-positive macrophages isolated from chronic wounds suggest that diabetes leads to a decrease in the anti-inflammatory macrophage type (40).

This macrophage proinflammatory transformation is attributed to a number of processes, the most important of which are oxidative stress and the production ROS. Advanced glycation end products (AGEs) are involved in the regulation of macrophage polarization (41). Some studies have shown macrophage dysfunction caused by the AGE/receptor for advanced glycation end products (RAGE) signalling axis. Compared with that in the normal control group, the number of M1-type macrophages was increased at the wound site in diabetic mice. Long-term hyperglycaemia in patients can increase the formation and accumulation of AGEs (41). AGEs can bind with RAGE to activate NADPH oxidase (NOX), induce oxidative stress, and promote the inflammatory response (42). A large number of studies have shown that AGEs are involved in the occurrence and development of DDs, but many researchers have focused on keratinocytes and fibroblasts (6). There have been few studies on AGEs and skin macrophages. However, it has been confirmed that AGEs can enhance the transformation of macrophages into the M1 type in other chronic complications associated with diabetes. It has been reported that oxidative stress is involved in the AGE/HIF-1α/PDK4 pathway to promote the development of atherosclerosis in individuals with diabetes mellitus (43).

In addition, our previous studies have shown changes in the composition and diversity of foot skin microbiota in diabetic patients, with an increase in the proportion of gram-negative bacteria and a decrease in the proportion of gram-positive bacteria (not yet published). LPS, the main component of the cell wall of gram-negative bacteria, can promote the secretion of TNF-α and IL-1 by macrophages by binding to the surface receptor TLR and stimulate the transformation of macrophages into the M1 type.

However, there have been some reports on the regulation of skin macrophage polarization in diabetic patients. However, the exact mechanism is still unclear. Further exploration will reveal the mechanism of diabetic skin inflammation and provide targets for its prevention.

### Impaired phagocytic function

If chemotactic movement is the premise of the phagocytic function of macrophages, then the phagocytic function of macrophages is the key to preventing the invasion of pathogens. As described above, the plasma and serum of diabetic patients contain a large number of chemokines, and the skin contains a large number of macrophages. It seems that when the skin is exposed to a pathogen, the body is fully prepared. Interestingly, however, the increased number of macrophages did not perform their normal phagocytosis. Liu et al. (38) confirmed that the phagocytosis of macrophages was impaired in a diabetic state in an animal model. This seems to explain why diabetic patients are prone to skin infections. Macrophages act as sentinels against skin infections. Studies have shown that the AGE/RAGE signalling axis in diabetic patients can impair the phagocytic function of macrophages. Ferracini et al. (44) and Rains and Jain (45) showed that impaired phagocytic function of macrophages in diabetic mice was related to insufficient coupling of the leukotriene (LT)/Fc γ R signalling cascade. Exogenous use of LTB4 and LTD4 can increase macrophage phagocytosis, which is conducive to killing pathogens.

### Abnormal necrotic function of macrophages

In addition to macrophage phagocytosis, the pathological status of diabetes also seems to affect the necrotic function of macrophages. Phagocytosis is the process by which macrophages remove foreign microorganisms, while necroptosis is the process by which macrophages remove apoptotic cells. Apoptosis is a noninflammatory form of cell death, and uptake is an important step for ensuring that these cells do not expand the inflammatory response. In mouse models of T2DM, the necrotic function of macrophages has been shown to be impaired (46). Macrophages are unable to successfully engulf apoptotic neutrophils and prevent the release of their toxic components, leading to further inflammatory responses. Excessive neutrophils cannot be phagocytosed by macrophages at the wound, and activated NETs can also activate NLRP3 inflammasomes through the TLR-4/TLR-9/NF-κB and ROS/TXNIP signalling pathways, further expanding inflammation and oxidative stress (44). This result suggests that the impairment of necrotic functions in diabetes may lead to macrophage pyroptotic death. Pyroptosis, a type of inflammatory cell death associated with NLRP3 inflammasomes, has been reported to play a role in diabetic skin ulcers. Future studies will reveal whether cell death or other cell death pathways are increased in diabetic macrophages and whether this mechanism contributes to the progression of diabetic complications.

### Epigenetic alterations regulate diabetic cutaneous macrophages

Epigenetic modification play an important role in diabetic skin lesions. Studies on DNA methylation, histone modification and non-coding RNA regulation in diabetic skin lesions (47–49) have been reported successively. Recently, Yan Et al. found that (50), N6-methyladenosine (m6A) RNA modification ultimately acts on skin wound healing by regulating the function of SQSTM1 in diabetic keratinocytes to regulate autophagy. New research suggests that epigenetic mechanisms regulate the polarization of macrophages to affect diabetic skin wound healing.

DNA methylation refers to the covalent binding of the cytosine (C) of the DNA template to the methyl group under the catalysis of methyltransferase (DNMT). Inhibition of DNMT1 has been reported to promote the formation of M2 macrophages (51). Under diabetic conditions, circulating hematopoietic stem cells (HSCs) are stimulated by oxidative stress, resulting in an upregulation of DNMT1 (52). In addition, studies confirmed that in diabetic mice, DNMT1 knockout initiates the production of M2 macrophages and improves insulin sensitivity and wound healing (51). Alterations in histone methylation have also been reported in macrophages of diabetic wounds. Absarmean et al. (53, 54) showed that monocyte MLL1 expression isolated from patients with type 2 diabetes mellitus increased, which regulates NF-κB-mediated transcription of inflammatory genes, reducing the production of pro-inflammatory factors leading to chronic wound non-healing. Histone H3 lysine-27 (H3K27) demethylase Jmjd3 is mainly associated with M1 polarization activation. BMDMs in diabetic models show enhanced activation of Jmjd3, affecting the overexpression of inflammatory IL-12 and leading to the onset of persistent wound inflammation (55, 56).

Taken together, these findings suggest that epigenetic abnormalities caused by hyperglycemia play an important role in regulating macrophage polarization in diabetic wounds. Epigenetic alterations are often heritable, influenced by environmental factors, and are reversible, providing optimistic prospects for the treatment of diabetic skin lesions.

## Deterioration of the inflammatory microenvironment: Cause or effect?

Abnormal activation of proinflammatory macrophages affects skin function, while hyperglycaemia induces various functional changes in skin macrophages. The interaction between pathological macrophage activation and metabolic disorders contributes to diabetic skin inflammation. As mentioned above, the inflammatory microenvironment of diabetes is caused by increased oxidative stress, impaired macrophage phagocytic functions, necrotic functions, and a tendency towards M1 polarization. Whether the inflammatory microenvironment of diabetes is the cause or effect of the abnormal activation of macrophages has not been well answered. This review is focused on this perspective, starting from the chronic inflammatory injury of macrophages, and explains the relationship. Normal skin tissues are damaged all the time, but there is no concern that the skin barrier will be damaged. Moreover, T2DM-associated inflammation is characterized by an increased number of macrophages in different tissues and the simultaneous production of the cytokines TNF-α, IL-1β, IL-6 and IL-8. This finding suggests that macrophages play a key role in the occurrence and maintenance of inflammation. In diabetes, the study of the inflammatory signalling pathway of macrophages is helpful for the prevention of inflammatory lesions in diabetic skin.

## Discussion

Past studies on skin macrophages in diabetic patients or diabetic animal models are scarce. However, thanks to advances in testing technology, skin inflammation can be examined much earlier. At present, increasing attention has been given to inflammation, and the function of macrophages has become a hot topic that cannot be ignored. Previous studies showed that the proinflammatory characteristics of macrophages in the diabetic state and the high levels of proinflammatory cytokines and chemokines are important markers of DD pathology. Although the precise molecular link between skin macrophages and the progression of DDs is unknown, studies suggest that macrophage regulation plays a positive role in the healing of diabetic ulcers. These findings suggest that novel therapies to reduce the inflammatory response of excessive skin macrophages have important clinical implications for the management of DDs. This consideration gives us insight that we should explore their occurrence and development from the early stage, as macrophages are an important cause of diabetic ulcers. For common skin diseases, it is best to study these cells from the perspective of pathology. However, DDs seem to be an insurmountable gap. Invasive tests have limited feasibility for patients with diabetes, since the skin is intact, so we urgently need to find a suitable alternative. The rise of noninvasive testing technology seems to solve this dilemma well.

## Data availability statement

Publicly available datasets were analyzed in this study. This data can be found here: https://www.ncbi.nlm.nih.gov/pmc/articles/PMC7733392/table/t0001/?report=objectonly.

## Authors contributions

JH contributed to the conception of the study. SZ contributed significantly to analysis and manuscript preparation. XD helped perform the analysis with constructive discussions. All authors contributed to the article and approved the submitted version.

## Funding

This work was supported by the National Natural Science Foundation of China (grant number 82170840).

## Conflict of interest

The authors declare that the research was conducted in the absence of any commercial or financial relationships that could be construed as a potential conflict of interest.

## Publisher’s note

All claims expressed in this article are solely those of the authors and do not necessarily represent those of their affiliated organizations, or those of the publisher, the editors and the reviewers. Any product that may be evaluated in this article, or claim that may be made by its manufacturer, is not guaranteed or endorsed by the publisher.
